# Biodegradation of Petroleum Sludge by *Methylobacterium* sp. Strain ZASH

**DOI:** 10.21315/tlsr2023.34.2.10

**Published:** 2023-07-21

**Authors:** Zakuan Azizi Shamsul Harumain, Mohd Azrul Naim Mohamad, Noor Faizul Hadry Nordin, Mohd Yunus Abd Shukor

**Affiliations:** 1 Department of Biotechnology, Kulliyyah of Science, International Islamic University Malaysia, 25200, Kuantan, Pahang, Malaysia; 2 Research Unit for Bioinformatics and Computational Biology, Kulliyyah of Science, International Islamic University Malaysia, 25200 Kuantan, Pahang, Malaysia; 3 International Institute for Halal Research and Training, International Islamic University Malaysia, 53100 Kuala Lumpur, Malaysia; 4 Department of Biochemistry, Faculty of Biotechnology and Science Biomolecule, Universiti Putra Malaysia, 43400 Serdang, Selangor, Malaysia

**Keywords:** Petroleum Sludge, Biodegradation Kinetics, Sawdust, *Methylobacterium* sp, Enapcemar Petroleum, Kinetik Biodegradasi, Habuk Papan, *Methylobacterium* sp

## Abstract

A bacterium was isolated from sludge-contaminated soil in a petroleum refinery and tested for its ability to degrade aliphatic hydrocarbon compounds present in petroleum sludge. The isolate was grown on minimal salt media agar supplemented with 1% (w/v) petroleum sludge. The isolate was tentatively identified as *Methylobacterium* s p. s t rain ZASH based on the partial 16s rDNA molecular phylogeny. The bacterium grew optimally between the temperatures of 30°C and 35°C, pH 7 and 7.5, 0.5% and 1.5% (v/v) Tween 80 as the surfactant, and between 1% and 2% (w/v) peptone as the nitrogen source. The constants derived from the Haldane equation were μmax = 0.039 hr^−1^, *K_s_* = 0.385% (w/v) total petroleum hydrocarbons (TPH) or 3,850 mg/L TPH, and *K_i_* =1.12% (w/v) TPH or 11,200 mg/L. The maximum biodegradation rate exhibited by this strain was 19 mg/L/hr at an initial TPH concentration of 10,000 mg/L. Gas chromatography analysis revealed that after 15 days the strain was able to degrade all aliphatic n-alkanes investigated with different efficiencies. Shorter n-alkanes were generally degraded more rapidly than longer n-alkanes with 90% removal for C-12 compared to only 30% removal for C-36. The addition of sawdust did not improve bacterial degradation of petroleum hydrocarbons, but it assisted in the removal of remaining undegraded hydrocarbons through adsorption.

## Abstrak

Bakteria telah diasingkan daripada tanah yang tercemar enap cemar dalam penapisan petroleum dan diuji untuk keupayaannya untuk merendahkan sebatian hidrokarbon alifatik yang terdapat dalam enap cemar petroleum. Pengasingan ditanam pada agar media garam minimum ditambah dengan 1% (b/v) enap cemar petroleum. Pengasingan telah dikenal pasti secara sementara sebagai *Methylobacterium* sp. terikan ZASH berdasarkan filogeni molekul rDNA 16s separa. Bakteria berkembang secara optimum antara suhu 30°C dan 35°C, pH 7 dan 7.5, 0.5 dan 1.5% (v/v) Tween 80 sebagai surfaktan, dan antara 1% dan 2% (b/v) pepton sebagai sumber nitrogen. Pemalar yang diperolehi daripada persamaan Haldane ialah μmax = 0.039 jam^−1^, *K_s_* = 0.385% (b/v) jumlah hidrokarbon petroleum (TPH) atau 3,850 mg/L TPH, dan *K_i_* = 1.12% (w/v) TPH atau 11,200 mg/L. Kadar biodegradasi maksimum yang ditunjukkan oleh strain ini ialah 19 mg/L/jam pada kepekatan TPH awal 10,000 mg/L. Analisis kromatografi gas mendedahkan bahawa selepas 15 hari terikan itu dapat merendahkan semua n-alkana alifatik yang disiasat dengan kecekapan yang berbeza. N-alkana yang lebih pendek secara amnya terdegradasi lebih cepat daripada n-alkana yang lebih panjang dengan penyingkiran 90% untuk C-12 berbanding dengan penyingkiran hanya 30% untuk C-36. Penambahan habuk papan tidak memperbaiki degradasi bakteria hidrokarbon petroleum, tetapi ia membantu dalam penyingkiran baki hidrokarbon yang tidak terdegradasi melalui penjerapan.

**Highlights**

- Isolation, characterisation, growth and the biodegradation kinetics of a novel strain of petroleum sludge-degrading *Methylobacterium* sp. strain ZASH.
- Showed that this strain has broad optimal temperature range, a trait that is useful in bioremediation.
- Provide new data on petroleum sludge-degrading biodegradation kinetics from *Methylobacterium* sp. not available in the literature from this genus.
- Provide new data on the effect of sawdust on petroleum sludge-degradation by this bacterium and discovered that it merely acts as an adsorption agent at least in liquid culture.

## INTRODUCTION

Petroleum production facilities such as refineries generate large quantities of petroleum sludge as a waste by-product. Petroleum refinery sludges have different characteristics depending on the crude feed and refining processes but generally the sludges are dark-thick-viscous-oily like products which consists of sediment, soil, water and high hydrocarbon concentration (Johnson & Affam 2019). As it is a waste by-product, the oily sludge warrants treatment and disposal to avoid groundwater contamination and other risks to human health and the environment. Existing methods to manage and treat this waste include storage, landfill disposal and incineration which are costly and generally not environmentally friendly (Islam 2015). Bioremediation of petroleum sludge is one of the several economic approaches that has gained global interest and recognition (Ossai *et al*. 2020). In the process of bioremediation of a pollutant, indigenous bacteria isolates is a preferential approach as the microorganisms are more tolerant to the local environmental and geographical conditions as well as posing lesser bureaucratic hurdles for permitted implementation, especially in Malaysia (Habib *et al*. 2018; Muliadi *et al*. 2021).

*Methylobacterium* sp. is one of the microbes often isolated from petroleum hydrocarbon-contaminated land (Srivastva *et al*. 2017; Yang *et al*. 2018). But, until recently, its potential for degrading petroleum hydrocarbon and growth and biodegradation kinetics on petroleum-sludge has not been studied. Sawdust has often been the bulking agent of choice for bioremediation of petroleum hydrocarbon-contaminated soil. The use of sawdust in biodegradation of petroleum sludge increases the hydrocarbon removal and degradation activity of the microbes by providing support material for bacterial growth and may also increase aeration rate (Huang *et al*. 2019; Ismail *et al*. 2019; Tanee & Jude 2017). The effect of sawdust on biodegradation of petroleum sludge by *Methylobacterium* sp. has not been studied. However, recent works have shown that sawdust had improved biodegradation rate of other pollutants such as benzophenone (Lin *et al*. 2021) and phenol (Abarian *et al*. 2019). In this work, we present data on the growth and biodegradation kinetics and the role of sawdust in petroleum sludge biodegradation by a strain of *Methylobacterium* sp. locally isolated from petroleum sludge.

## MATERIALS AND METHODS

### Isolation and Screening of Bacteria

Petroleum sludge samples were collected at the Petronas Refinery Centre in Kerteh, Terengganu, Malaysia. The samples were collected from the effluent treatment plant in sterile polypropylene containers. Ten grams of sludge was inoculated into 100 mL of sterile 0.1% phosphate buffer saline in a 250 mL conical flask, and incubated at 30°C with shaking at 150 rpm for 10 days (Ali *et al*. 2011). Then, 8-fold serial dilutions were carried out and 0.1 mL of 10^−8^ dilutions was spread onto nutrient agar. The isolated bacteria were cultured again on minimal salt media supplemented with 0.1 g/L of petroleum sludge (AL-Doury 2019). The composition of the medium (g/L) was as follows: 4.0 Na_2_HPO_4_, 2.0 KH_2_PO_4_, 0.8 NH_4_SO_4_, 0.8 MgSO_4_ and 1 mL of trace element solution (TES) per litre. TES contained (g/L) 0.1 Al(OH)_3_, 0.05 SnCl_2_.2H_2_O, 0.05 KI, 0.05 LiCl, 0.08 MgSO_4_.4H_2_O, 0.5 H_3_BO_3_, 0.1 ZnSO_4_.7H_2_O, 0.1 CoCl_2_.6H_2_O, 0.1 NiSO_4_.6H_2_O, 0.05 BaCl_2_ and 0.05 m/L (NH_4_). This media was supplemented with 1% (w/v) of petroleum sludge in 100 mL of media. For solid medium, 20 g of bacteriological agar was added to solidify the medium. The sludge was dissolved using diethyl ether and evenly distributed onto the agar to provide a thin layer of petroleum oily sludge (Xu *et al*. 2019). The plates were then incubated at room temperature for 21 days. After 21 days, several positive colonies were obtained. The best isolate-strain ZASH, based on bacterial growth measurement at OD_600nm_ on liquid media, was chosen for further studies. The bacterium was maintained on agar slant at 4°C for routine use or stored at −20°C in Microbank^TM^ vials for long-term storage.

### Petroleum Sludge Analysis

The moisture content of the petroleum sludge collected was determined by oven drying overnight at 105°C (Y. Wang *et al*. 2017). The amount of moisture was determined by: (weight of beaker + sludge before dry) − (weight of beaker + sludge after dry). This method was performed in triplicate to obtain the amount of moisture present in the petroleum sludge.

### Total Petroleum Hydrocarbon Fractionation

The total petroleum hydrocarbons (TPH) fraction of the petroleum sludge was extracted (Suganthi *et al*. 2018). About 1 g of air-dried petroleum sludge was extracted with 100 mL of hexane, dichloromethane, and chloroform, successively and was air dried to evaporate the solvent. Extracted TPH at about 0.2 g to 0.5 g, were dissolved in n-pentane and were separated into soluble and insoluble fractions. Soluble fractions were loaded onto a silica gel column. Alkane fraction was eluted with the addition of 100 mL of hexane, followed by the elution of the aromatic fraction by the addition of 100 mL of benzene. The resins fraction was eluted with the addition of a mixture of chloroform and methanol (100 mL each). After evaporation, the extracted fractions were determined gravimetrically (G Wang *et al*. 2017). These analyses were performed in triplicate.

### Identification of Petroleum Sludge-Degrading Bacterium

Identification of the isolated strain was based on morphological characteristics through microscopic observation, Gram-reaction, spore staining, biochemical tests and molecular phylogenetic analysis. For routine use, the strain was maintained on nutrient agar slant at 4°C. For long-term storage, the strain was preserved in 70% of sterile glycerol (v/v) at −20°C.

### 16S rRNA

DNA was extracted through alkaline lysis using the DNeasy® Blood and Tissue Purification Kit^TM^ (Qiagen, USA) according to the manufacturer’s instructions. Amplification of the 16s rRNA gene was performed using the universal primers; 5′-AGAGTTTGATCATGGCTCAG-3′ and 5′-ACGGTTACCTTGTTACGACTT-3′ synthesized by 1st Base Sdn. Bhd. (Malaysia) corresponding to the forward and reverse primers of 16S rRNA, respectively (Lee *et al*. 2018). The PCR reaction mixture consisted of 43 μL sterilised distilled water, 2 μL of PCR buffer (10x) (Fermentas, USA), 2 μL of MgCl_2_ (2 mM) (Fermentas, USA), 0.5 μL deoxynucleoside triphosphates (dNTP) (10 mM) (Fermentas, USA), 0.5 μL (100 μM) of each primer solution, 1 μL of DNA template solution, and 0.5 μL of Taq DNA polymerase (Qiagen, USA). PCR was performed (T-Gradient thermocycler, Biometra, Germany) under the following conditions: initial denaturation at 94°C for 4 min, 30 cycles of denaturation (1 min at 94°C), annealing (2 mins at 58°C); followed by final extension at 72°C for 10 min. Purification of the PCR products was carried out by centrifugation using the Wizard SV Gel and PCR Clean-Up System (Promega, Madison, USA). The purified PCR product was sent for automated fluorescent sequencing. The 1,439 bases obtained were compared with the GenBank database using the NCBI Blast server (http://blast.ncbi.nlm.nih.gov/Blast.cgi). The partial 16S rRNA ribosomal gene sequence for this isolate have been deposited in GenBank under the accession number JN850063.

### Phylogenetic Analysis

A phylogenetic tree was constructed by using PHYLIP, version 3.573 (Felsenstein 1985), with *Bacillus* as the outgroup in the cladogram. A multiple alignment of 19 16S rRNA gene sequences closely matches strain ZASH were retrieved from GenBank and were aligned using Clustal Omega. The output option was PHYLIP format. Gaps within the alignment were excluded from calculations (Thompson *et al*. 1994). The suite of programmes from PHYLIP used in sequence were SEQBOOT, DNADIST, NEIGHBOR and CONSENSE. The confidence levels for individual branches to be generated within the tree were carried out with 1,000 bootstraps (Felsenstein 1985) by the SEQBOOT program in the PHYLIP package. Evolutionary distance matrices for the neighbour-joining/UPGMA methodology were then computed using the DNADIST algorithm programme. The programme reads in nucleotide sequences and writes an output file containing the distance matrix. The model of nucleotide substitution is those of Jukes and Cantor (Jukes & Cantor 1969). The phylogenetic tree was inferred by using the neighbour-joining method of Saitou and Nei (Saitou & Nei 1987) in the NEIGHBOR programme. Majority rule (50%) consensus trees were constructed for the topologies found using a family of consensus tree methods called the Ml methods (Margush & McMorris 1981) using the CONSENSE program and the tree was viewed using TreeView (Page 1996).

### Characterisation Studies

The ability of the strain to grow and degrade hydrocarbons in petroleum sludge was characterised according to its effect on different nitrogen sources and concentration, surfactant sources and concentration, pH, temperature, and carbon concentration. The growth was determined by performing colony forming unit (CFU) method and degradation studies were quantified using gas chromatography with a flame ionisation detector.

### Effect of Sawdust on Petroleum Sludge Degradation

Sawdust was obtained from a wood factory (Woodland Resources Sdn. Bhd.) in Kepong, Selangor, Malaysia. Sawdust comes in various size and shape. In this test, the size of sawdust used was 0.3 mm and separated using a sieve shaker. Sawdust was autoclaved before use. Different concentrations of sawdust were used starting from 1%, 2%, 3%, 4% and 5% (w/v) of sawdust and were added into 100 mL of minimal salt media containing 1% (w/v) of petroleum sludge. The media was then inoculated with 1 mL of bacterial culture (OD_600nm_ = 0.7–0.8) and incubated on an orbital shaker (YIHDER, Taiwan) at 150 rpm. This media was supplemented with 1% (v/v) of Tween 80 as a surfactant and 1.5% (w/v) of peptone as the nitrogen source. Bacterial addition was omitted in the control experiment. After 15 days, the concentration of aliphatic petroleum sludge was measured using gas chromatography with a flame ionization detector (GC-FID) (Behera *et al*. 2020).

The amount of petroleum sludge degraded by *Methylobacterium* sp. strain ZASH with the presence of 5% sawdust was determined by the equation as follows:


$$\begin{array}{l} \text{Amount of TPH degraded by }Methylobacterium\;\text{sp.}\;\text{strain ZASH}\\ \text{with the addition of }5\%\;\text{sawdust}=(\text{TPH loss in media with sawdust}\\ \text{and bacteria})-(\text{TPH loss in media containing sawdust}) \end{array}$$

### Gas Chromatography Analysis

The residual petroleum hydrocarbons in culture media were extracted with an equal volume of hexane. The organic phase was separated from the aqueous phase using a low-speed centrifuge. Anhydrous sodium sulfate was added to absorb residual water. The extracts were analysed by a gas chromatograph (GC model Agilent Technologies 7890A) equipped with a flame ionisation detector and a capillary column (TC-1, 30 m × 320 μm × 0.25 μm; film thickness = 0.1 μm) (J&W Scientific). Helium was used as carrier gas and was set at a constant flow rate of 2 mL/min. Injector and detector temperatures were 280°C and 300°C, respectively. The sample was initially held at 60°C for 6 min and then heated to 300°C at rate of 15°C min^−1^ where it was held for 20 min. The percentage of TPH degraded was calculated by the method of (Xia *et al*. 2019). All experiments were carried out in triplicate.

### Statistical Analysis

The data were generated in triplicate and analysed using Graphpad Prism version 3.0. Values shown are means ± SE. Student’s *t*-test or a one-way analysis of variance with post hoc analysis by Tukey’s test was used in evaluating statistical significance between groups.

## RESULTS

### Petroleum Sludge Analysis

**Figure 1:**
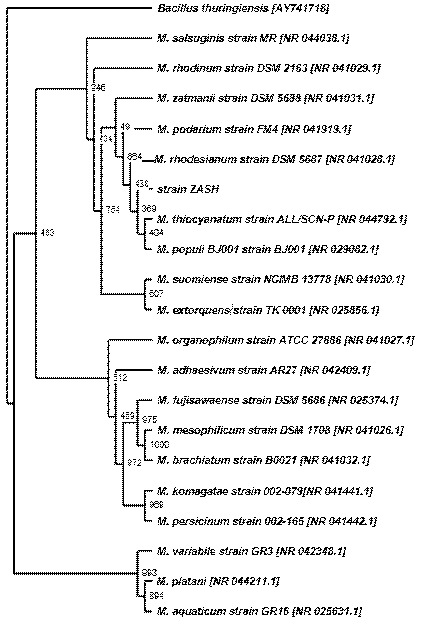
Neighbour-joining method cladogram showing phylogenetic relationship between strain ZASH and other related *Methylobacterium* sp. based on the 16S rRNA gene sequence analysis. Species names are followed by the accession numbers of their 16S rDNA sequences. The numbers at branching points refer to bootstrap values based on 1,000 re-samplings. The scale bar represents 100 nucleotide substitutions. *Bacillus thuringiensis* sp. is the outgroup.

The petroleum sludge obtained had a black to dark green clay-like appearance. The moisture content was 1.12% and the pH was 7.12. One gram of petroleum sludge consisted of about 0.71 g of TPH. The petroleum hydrocarbon fractions which can be obtained from the petroleum sludge through selective extractions are shown in Table 1. The highest fraction was n-alkanes followed by asphaltenes, aromatics, and nitrogen sulfur oxygen (NSO) resins. A petroleum sludge-degrading Gram-negative bacterium was successfully isolated from petroleum sludge samples taken from the Shell refinery centre sludge. The colonies were convex and pink while light microscopy showed a rod-shaped like bacterium. Identification of the bacterium was carried out based on the 16S rRNA gene sequence of the bacterium. Genetic analysis showed a moderate bootstrap value (36.9%) linking strain ZASH to the clade harbouring *M. rhodesianum* and to *M. thiocyanatum* and *M. populi* indicating that strain ZASH could not be tied to any of these species (Fig. 1). Hence, strain ZASH is tentatively identified as *Methylobacterium* sp. strain ZASH. The bacterium is mesophilic with a broad optimal range for both growth and degradation. The bacterium is suitable for bioremediation programmes in a tropical climate.

### Optimum Temperature

**Figure 2:**
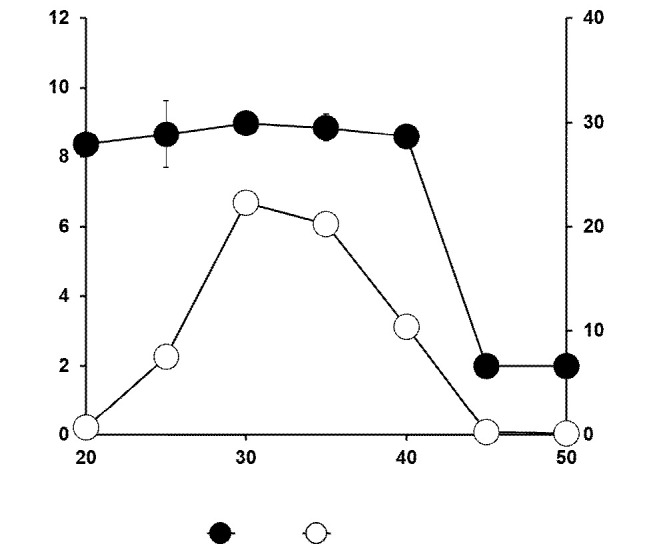
Effect of temperature on the biodegradation of petroleum sludge (%) and growth of *Methylobacterium* sp. strain ZASH (CFU/mL). The initial concentration of petroleum sludge was 1% (w/v). Each point represents the mean of triplicates ± SD.

Although bacterial growth showed no significant difference (*p* > 0.05) over a range of temperatures from 20°C to 40°C, hydrocarbon degradation was the highest at 30°C. Complete abolishment of degradation activity occurred at temperatures 20°C and below, and 45°C and higher (Fig. 2).

### Optimum pH

**Figure 3:**
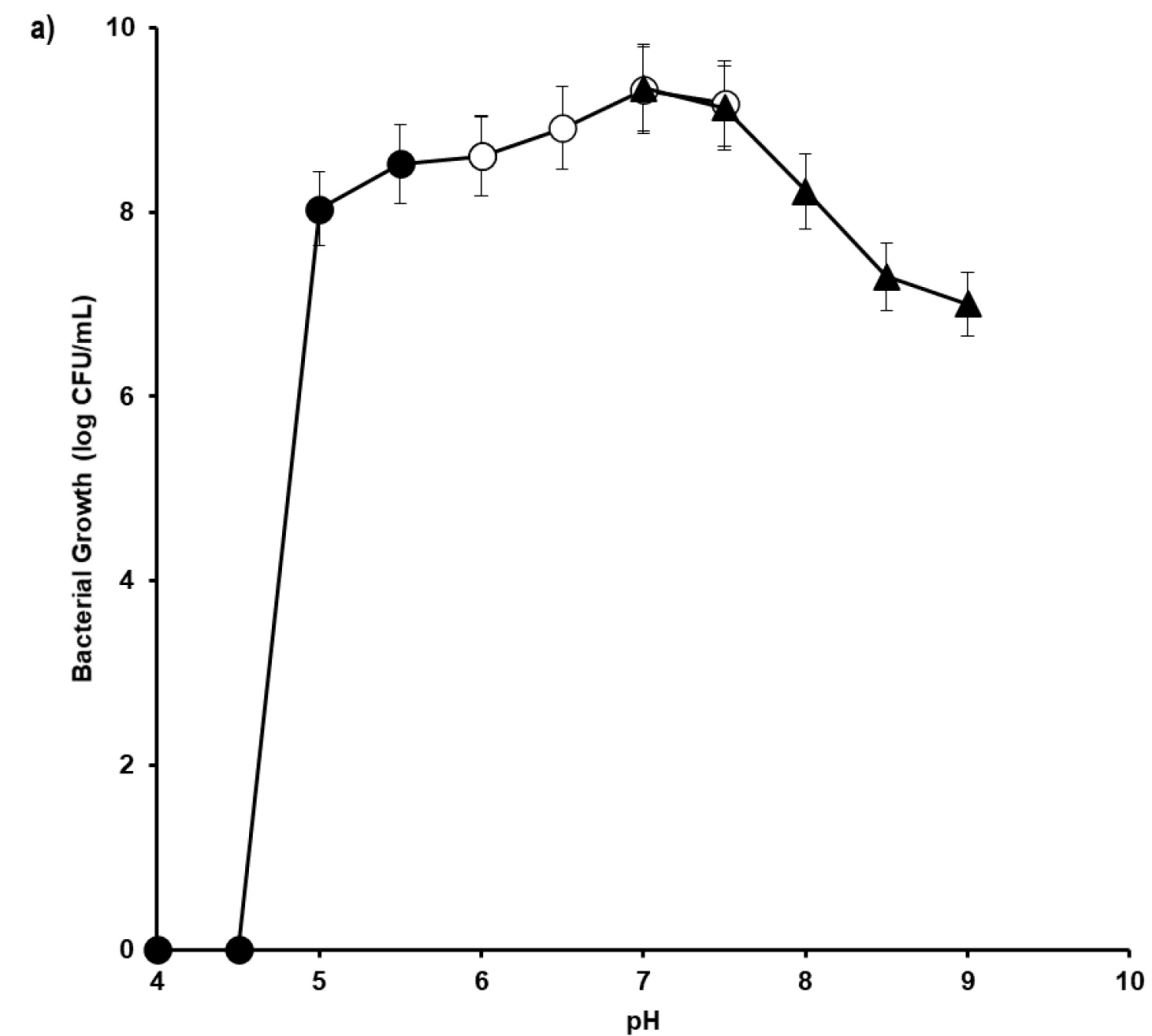

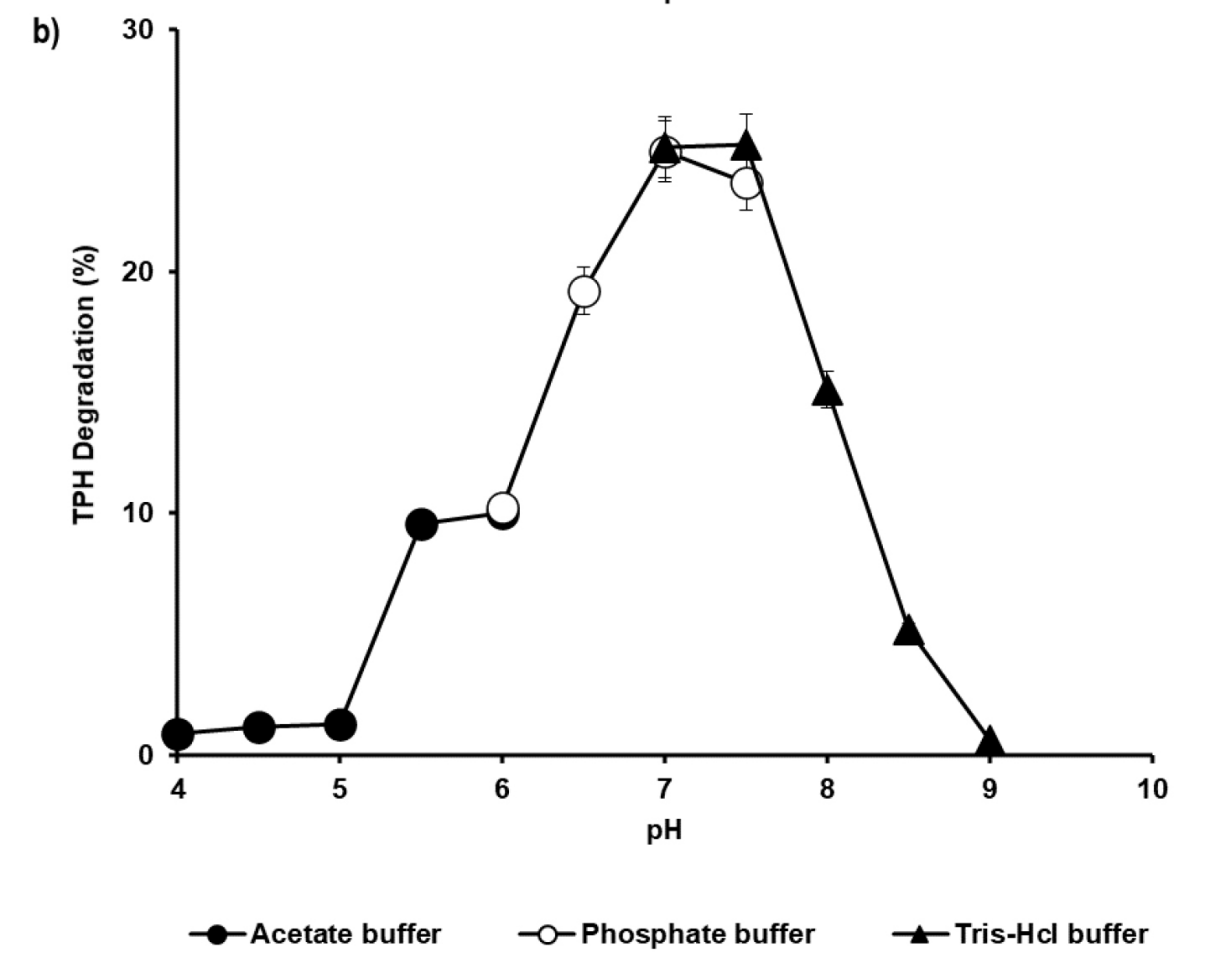
Effect of pH on the growth (CFU/mL) (a) and biodegradation of petroleum sludge (%) (b) by *Methylobacterium* sp. strain ZASH. The initial concentration of petroleum sludge was 1% (w/v). Each point represents the mean of triplicates ± SD.

In this study, an overlapping buffer system consisting of acetate buffer (4, 4.5, 5, 5.5 and 6), phosphate (6, 6.5 and 7), and Tris-HCl (7, 7.5, 8, 8.5 and 9) was used. The results showed that both bacterial growth and degradation were extremely sensitive to pH with complete inhibition of both activities occurring at pH lower than 4.25 and higher than 9.0. Degradation was optimal pHs between 6.25 and 8.0 with no significant difference (*p* > 0.05) in terms of degradation at pHs within this range. Growth, however, was optimal at a narrower range between 7.0 and 7.5 (Fig. 3).

### Effects of Nitrogen Source

**Figure 4:**
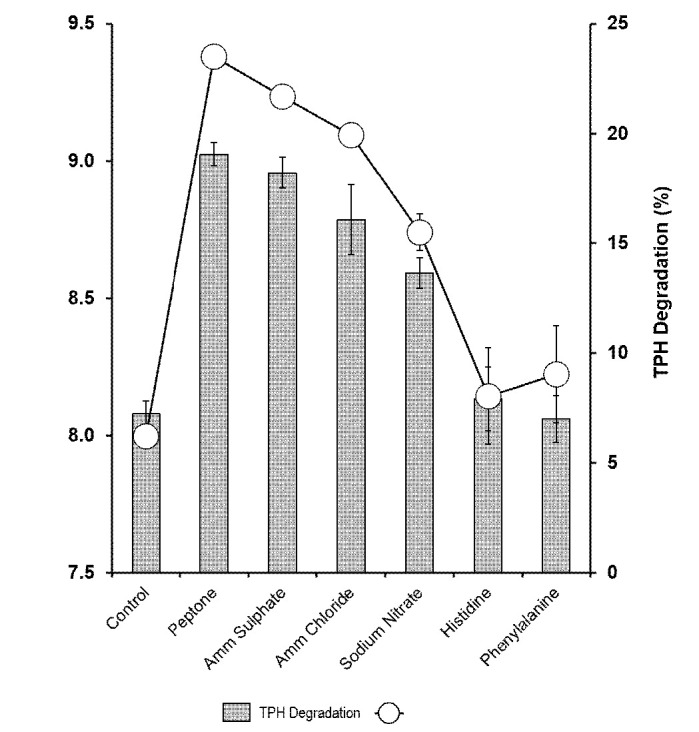
Effect of nitrogen sources on the biodegradation of petroleum sludge (%) and growth of *Methylobacterium* sp. strain ZASH (CFU/mL). Control used was without addition of nitrogen source. The initial concentration of petroleum sludge was 1% (w/v). Each point represents the mean of triplicate ± SD.

**Figure 5:**
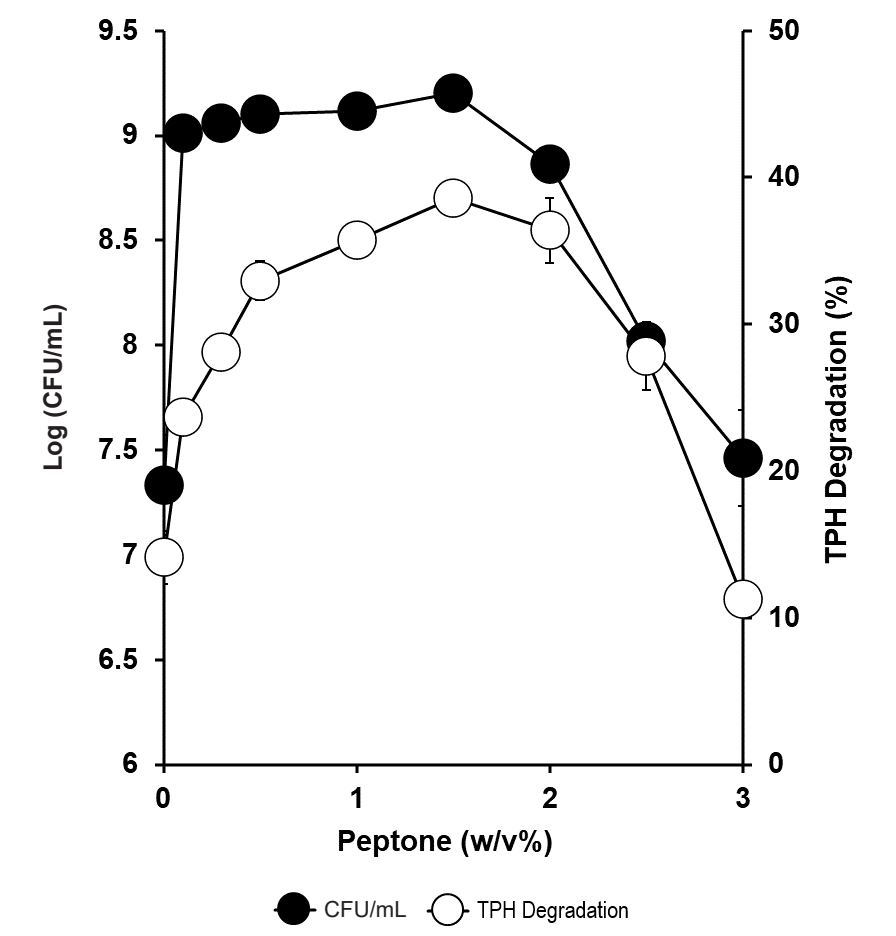
Effect of various concentrations of peptone on the biodegradation of petroleum sludge (%) and growth of *Methylobacterium* sp. strain ZASH (CFU/mL). The initial concentration of petroleum sludge was 1% (w/v). Each point represents the mean of triplicates ± SD.

In this study, inorganic and organic nitrogen source were added to the minimal salt media to investigate its effect on biodegradation of aliphatic hydrocarbons in petroleum sludge by *Methylobacterium* sp. strain ZASH. Peptone, ammonium sulphate, ammonium chloride and sodium chloride, all equally supported strain ZASH growth with no significant difference in terms of bacterial growth among them. However, peptone supported significantly (*p* < 0.05) the highest hydrocarbon degradation. Both histidine and phenylalanine did not significantly increase degradation and bacterial growth compared to the control (Fig. 4). Degradation activity was optimal between 1% and 2% (w/v) peptone with no significant difference between the concentrations (*p* > 0.05). Degradation decreased rapidly at higher peptone concentrations (Fig. 5).

### Effects of Surfactants

**Figure 6:**
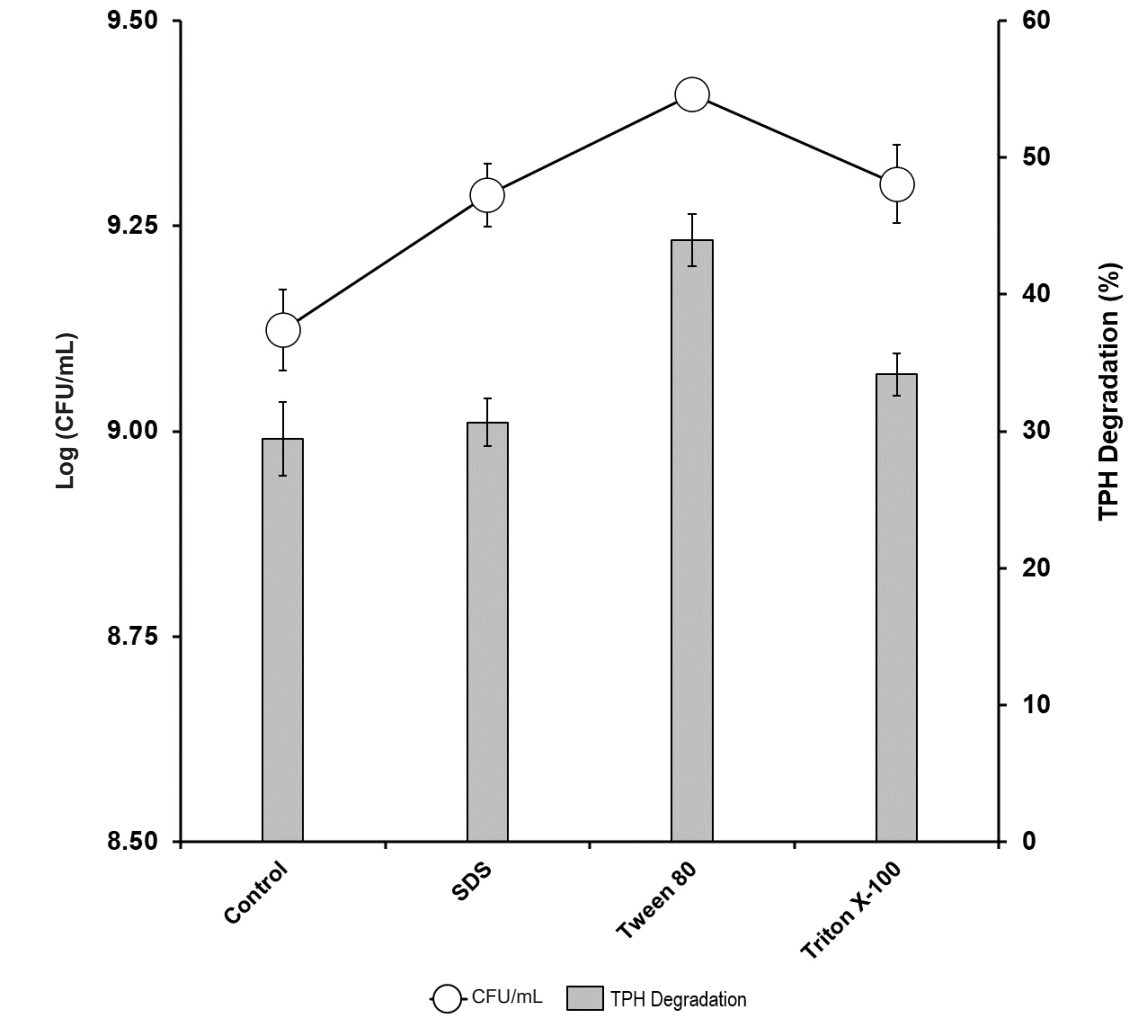
Effect of various surfactants at 0.1% (w/v) on the biodegradation of petroleum sludge (%) and growth of *Methylobacterium* sp. strain ZASH (CFU/mL). The initial concentration of petroleum sludge was 1% (w/v). Each point represents the mean of triplicates ± SD.

**Figure 7:**
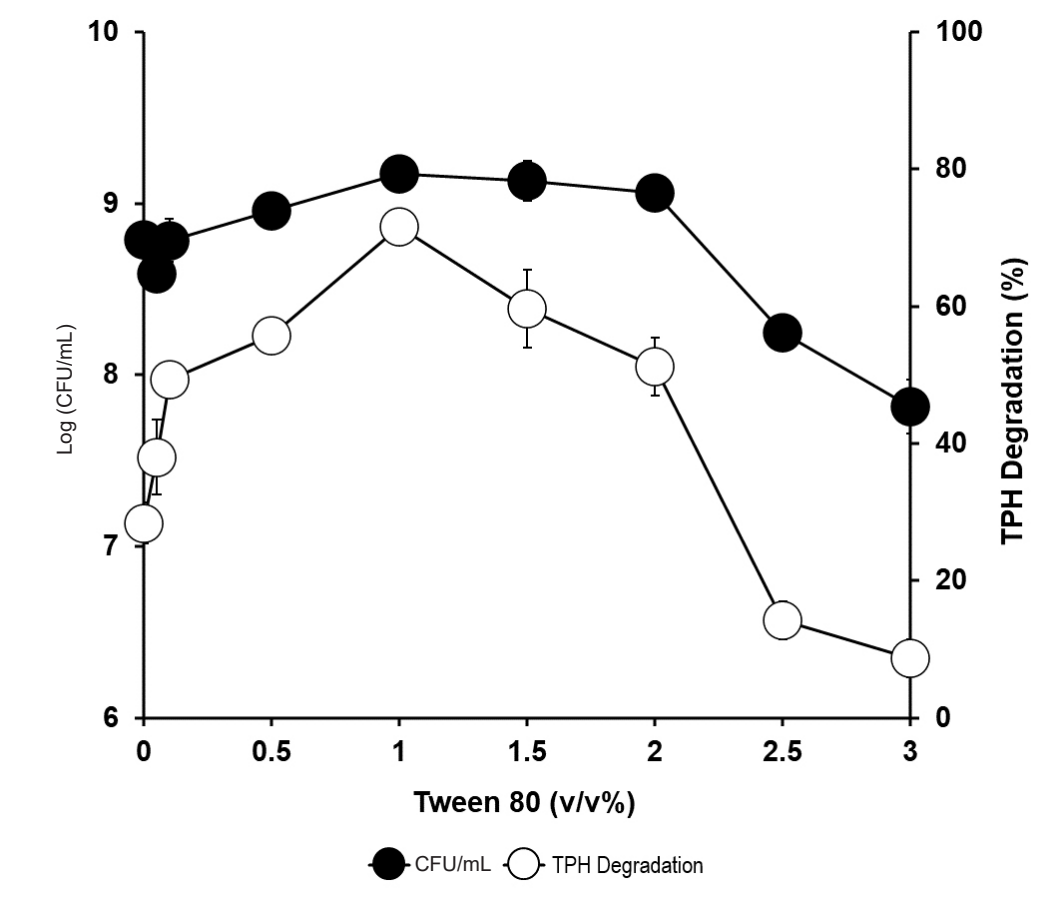
Effect of various concentrations of Tween 80 on the biodegradation of petroleum sludge (%) and growth of *Methylobacterium* sp. strain ZASH (CFU/mL). The initial concentration of petroleum sludge was 1% (w/v). Each point represents the mean of triplicates ± SD.

The effect of surfactant was studied using three different surfactants (Tween 80, sodium dodecyl sulfate [SDS] and Triton X-100) at 0.1% (w/v). Only Tween 80 showed enhanced growth on petroleum sludge. An increase in degradation was observed upon the addition of all surfactants but Tween 80 supported the highest degradation (Fig. 6). The optimal concentration of Tween 80 was 1%. Growth significantly increased compared to the control without surfactant with the highest tolerable Tween 80 concentration of 2%. Both growth and degradation were inhibited at higher Tween 80 concentrations (Fig. 7).

### Effects of Carbon Concentration

**Figure 8:**
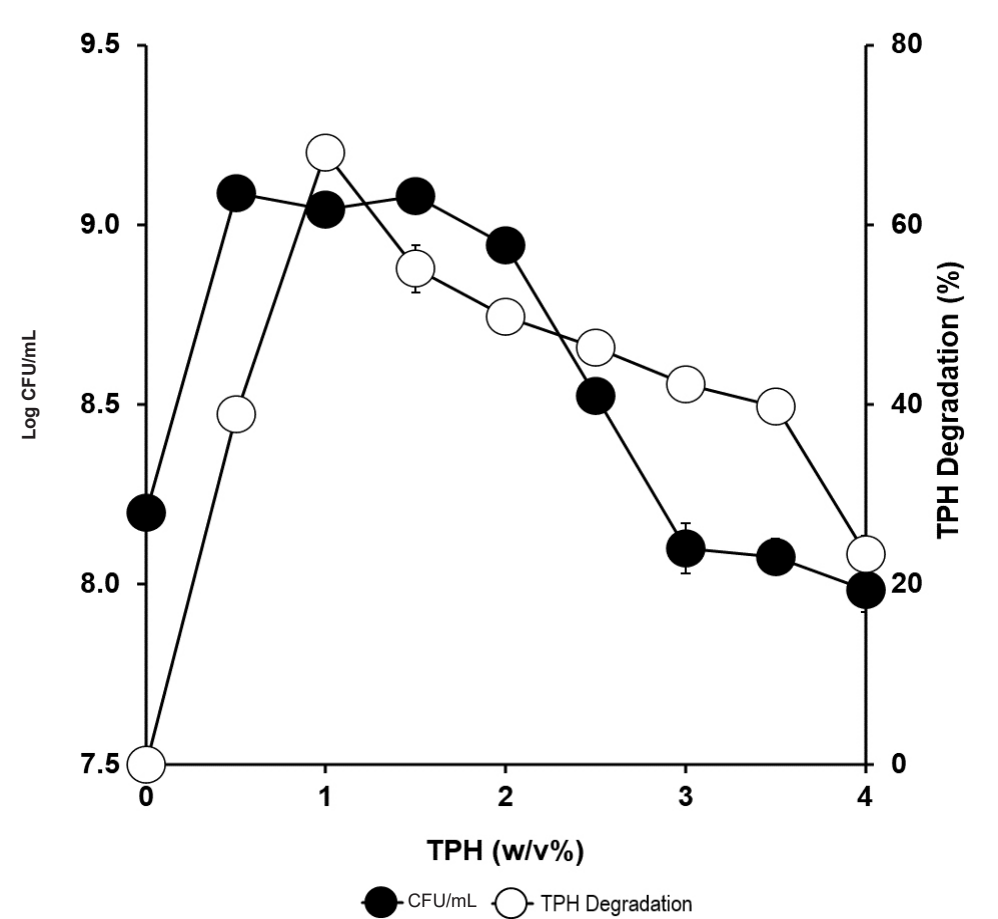
Effect of various concentrations of petroleum sludge as a carbon source on biodegradation (%) and growth of *Methylobacterium* sp. strain ZASH (CFU/mL). The initial concentration of petroleum sludge was 1% (w/v). Each point represents the mean of triplicates ± SD.

The effect of petroleum sludge concentration on growth and degradation was investigated. Degradation was optimal at 1% hydrocarbons and was somewhat inhibited at higher concentrations. About 20% degradation was observed at the highest tolerable concentration of petroleum sludge at 3%. Growth was optimally supported by hydrocarbon concentrations between 0.5% and 1.5% and was inhibited at higher concentrations. Degradation was observed at hydrocarbon concentrations higher than 3% although growth was abolished (Fig. 8).

### Biodegradation Profiles

**Figure 9:**
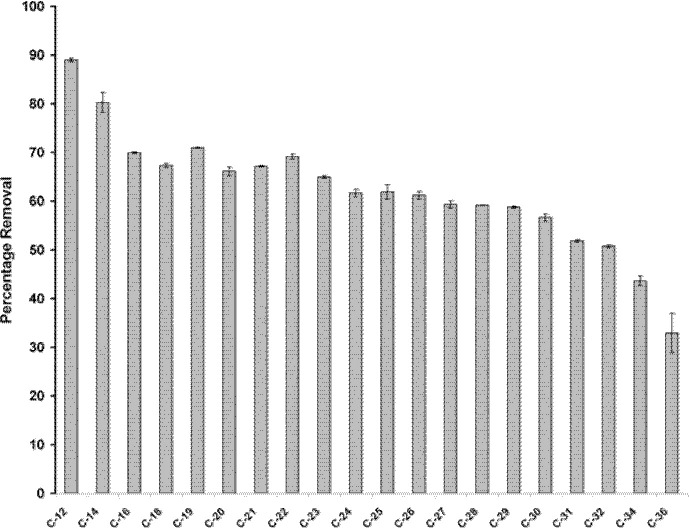
Degradation profile of the carbon component of petroleum sludge by *Methylobacterium* sp. strain ZASH after 15 days incubation. The initial concentration of petroleum sludge was 1% (w/v). Each point represents the mean of triplicates ± SD.

Biodegradation profiles of the assimilable major aliphatic components of hydrocarbon compounds involved in the process were quantified using gas chromatography analysis. The peaks obtained were then identified using a set of n-hydrocarbon mix standards obtained from Supelco® (USA) by matching the retention time of the eluting chromatographic peaks for the sample with the standards. The percentage removal of each aliphatic component of the petroleum sludge is shown in Fig. 9. The results have been adjusted using the results of the abiotic control for each of the aliphatic n-alkanes. *Methylobacterium* sp. ZASH degraded all the aliphatic long chain n-alkanes investigated with different efficiencies. Shorter n-alkanes are generally preferred than longer n-alkanes with 90% removal for C-12 compared to only 30% removal for C-36.

### Growth and Degradation Kinetics

**Figure 10:**
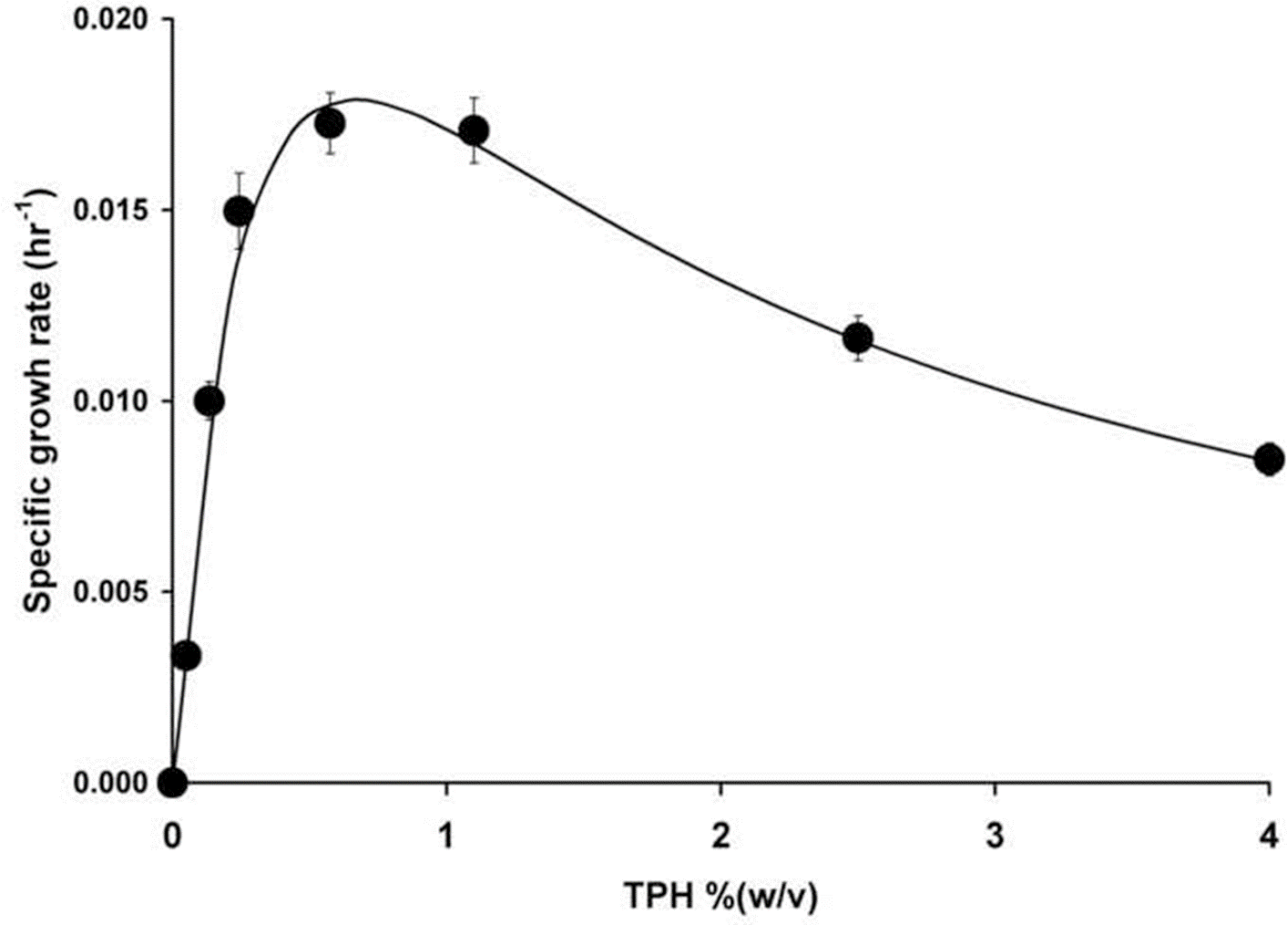
Maximum specific growth rates for TPH concentrations ranging from 0% to 4% (w/v). Each data point represents mean ± SD (*n* = 3).

The calculated specific growth rate for each TPH concentration and the slope of the linear logarithmic plot of optical density (OD 600 nm) against time was constructed. The slope was then plotted against substrate concentration (Fig. 10). The Haldane model or Andrews model (Monod with inhibition) was used to find the value for the maximum growth rate (μmax), the inhibition constant *K_i_*, and the half saturation constant *K_s_*. The data fitted the Haldane’s model producing a curve with a correlation coefficient of 0.983. The constants derived from the Haldane equation were μmax = 0.039 hr^−1^, *K_s_* = 0.385% (w/v) TPH or 3,850 mg/L TPH, and *K_i_* =1.12% (w/v) TPH or 11,200 mg/L. The biodegradation rate exhibited by this strain increases with an increase in initial TPH concentration reaching a maximum degradation rate of 19 mg/L hr^−1^ at an initial TPH concentration of 10,000 mg/L. An appreciable level of degradation rate of 4.1 mg/L hr^−1^ was observed at the initial TPH concentration of 40,000 mg/L or 4% (w/v) even though growth was completely abolished at this concentration as shown in Fig. 8 previously.[Fig f11-tlsr-34-2-197]

### Effects of Sawdust

**Figure 12:**
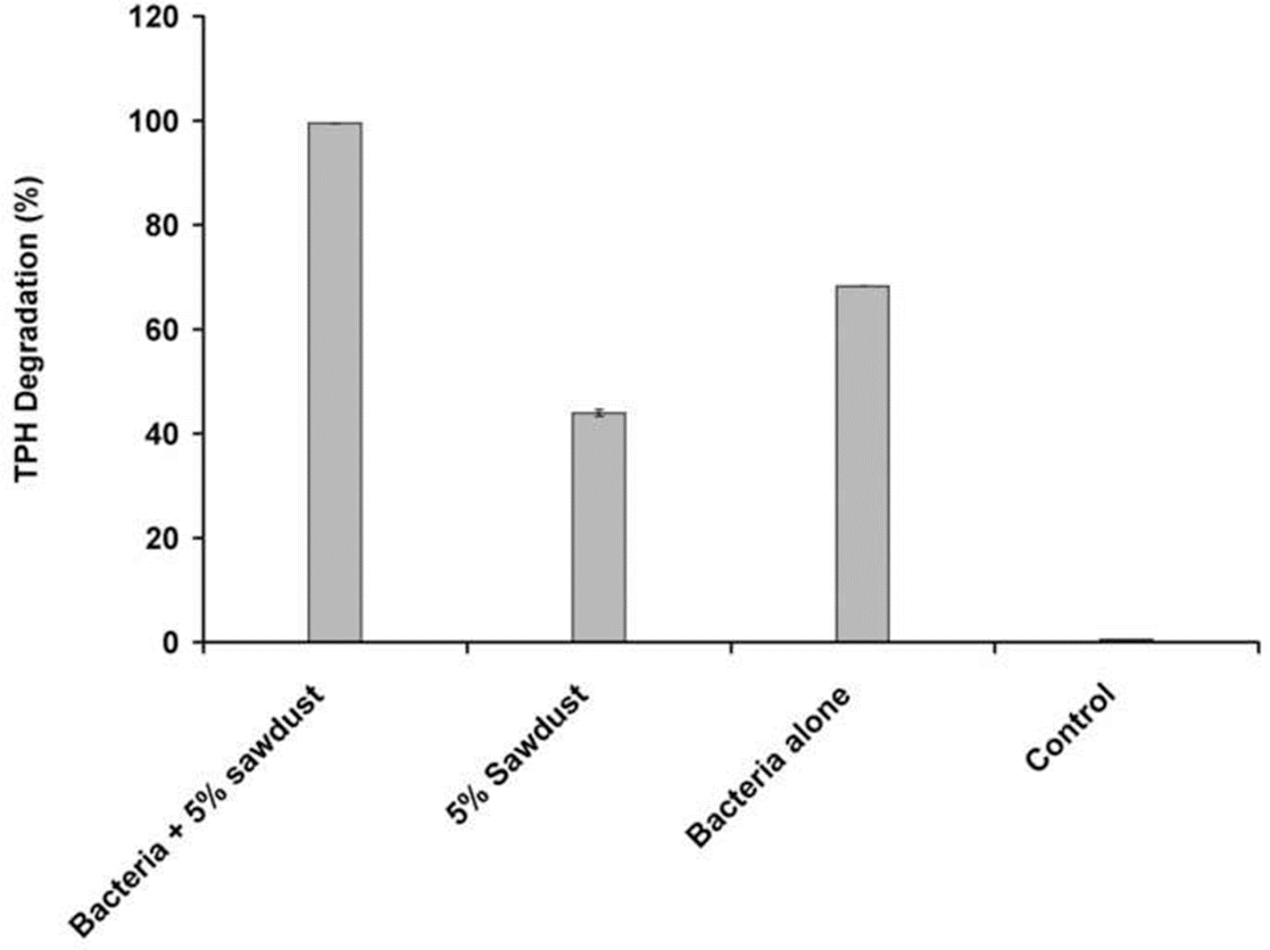
Effect of various concentrations of sawdust on aliphatic hydrocarbon degradation and cell density (growth) of *Methylobacterium* sp. strain ZASH. Control used was without bacteria or sawdust. Each point represents the mean of triplicates ± SD.

**Figure 13:**
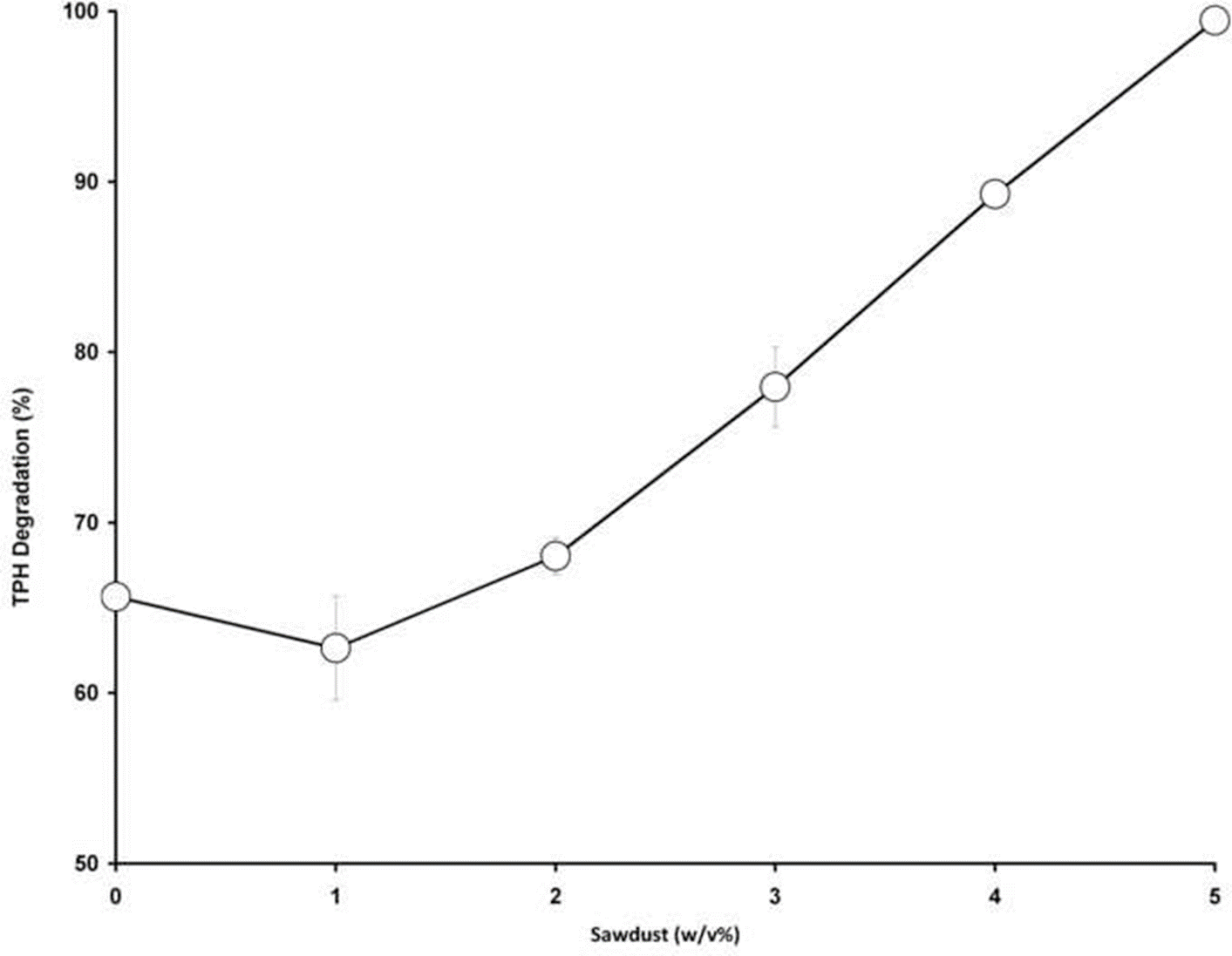
Effect of sawdust addition on hydrocarbon degradation of *Methylobacterium* sp. strain ZASH. Each point represents the mean of triplicates ± SD.

Sawdust is a waste product which consists of lignin, cellulose and hemicelullose. It is well known for its absorption characteristic and widely used to remove spilled oil (Huang *et al*. 2019; Ismail *et al*. 2019). The addition of sawdust appears at first to generally improve hydrocarbon degradation. Complete hydrocarbon degradation was observed at 5% sawdust (Fig. 12). The effect of sawdust as a hydrocarbon adsorption agent was investigated to elucidate the role of sawdust addition to the efficiency of degradation. It was discovered that sawdust merely acts as an absorption agent with 5% sawdust adsorbing nearly 40% of the 1% (w/v) petroleum sludge added into the media while strain ZASH removes 68% of the hydrocarbon (Fig. 13).

## DISCUSSION

Many hydrocarbon-degraders reported in the literature have a limited range of optimum temperatures supporting growth (D. Wang *et al*. 2019; Xia *et al*. 2019). Only a few have broad optimal temperature range of hydrocarbon-degrading activity suitable for wide-ranging geographical applications. On the other extreme, the psychrotolerant *Acinetobacter* sp. and *Pseudomonas aeruginosa* have been shown to degrade hydrocarbons at the optimal temperature of 5°C and 20°C, respectively (Wongsa *et al*. 2004), while thermophilic bacteria (*Pseudomonas aeruginosa and Bacillus thermosaudia*) have also been used to effectively degrade petroleum hydrocarbons at the optimum temperature of 60°C (Pugazhendi *et al*. 2017). Since soils in Malaysia could reach temperatures as high as 35°C (Sabullah *et al*. 2017; Shukor *et al*. 2009), this isolate is a suitable indigenous bacterium that could be employed in the bioremediation of hydrocarbons, both locally and in other tropical regions.

The optimal pH supporting growth of the strain is within the range of pH often found in petroleum sludge from 6 to 7.5 (Alhefeiti *et al*. 2021; Cheng *et al*. 2017; Devi *et al*. 2011; Hamidi *et al*. 2021; Rahman *et al*. 2007; Yan *et al*. 2012) indicating bioremediation programmes could be carried out without the addition of pH-adjusting chemicals. According to Shuler and Kargi (1992), pH will affect the cellular function, cell membrane and protein transport. Extremes in pH may invite a negative influence towards the ability of microbes to degrade hydrocarbons (Mishra *et al*. 2001; Nikitina *et al*. 2003). The study of pH optimum for the degradation of hydrocarbon in petroleum sludge would be highly useful for bioremediation purposes, especially when geographical locations of contaminated sites often dictate the type of suitable microbes to be used.

Nitrogen is important in enhancing *in situ* bioremediation. As one of the major nutrient sources for bacteria, the ratio of nitrogen to the carbon and phosphorus sources should be maintain at about 10:120:1 to sustain microbial activity (Roleda & Hurd 2019). Peptone has been found to increase the growth and degradation rate of petroleum hydrocarbons (Hamzah *et al*. 2013; Ji *et al*. 2019). Peptone consists of peptide and can be obtained from animal milk by proteolytic digestion. The bacterium will usually utilise easily assimilable compounds like peptone for growth and upon the depletion of peptone will then assimilate hydrocarbons (Hamzah *et al*. 2012; 2013).

Surfactants are usually added to increase the bioavailability of hydrocarbons by producing smaller hydrocarbon globules that are more easily attacked by microbes (Cecotti *et al*. 2018; Shivanand *et al*. 2021). The addition of surfactant below the critical micelle concentration (CMC) will increase the bioavailability of hydrophobic components (Shaban & Kim 2020; G. Wang *et al*. 2017). Tween 80 can stimulate the growth and degradation of hydrocarbons only in a limited range of concentration especially below the critical micelle concentration (CMC) because of its toxicity (Effendi *et al*. 2017; Saeedi *et al*. 2019; Somtrakoon & Chouychai 2019). The highest Tween 80 concentration that the *Methylobacterium* sp. ZASH tolerated is 2.5%. Other than that, surfactant may also reduce the rate of bioremediation due to increased toxicity caused by the increased solubility of toxic hydrophobic compounds in petroleum sludge (Kaczorek *et al*. 2018). The inhibitory effect of high concentrations of petroleum sludge could also be due to heavy metals (Sarkar *et al*. 2016). Usually diluting agents such as sawdust (Huang *et al*. 2019), zeolite (Zhao *et al*. 2018), and straw (Chen *et al*. 2019) are added to decrease petroleum sludge toxicity and at the same time to increase aeration.

Alkanes with lower carbon number (C-10) are the most preferable substrate for microbial degradation as it is more susceptible for microbial attack and, thus, more readily degraded (Liu *et al*. 2020). The priority of the microorganisms to attack hydrocarbon can be ranked as follows: linear alkanes > branched alkanes > small aromatics > cyclic alkanes > high molecular weight aromatics (Liu *et al*. 2020). The ability of *Methylobacterium* sp. ZASH to degrade long chain n*-*alkanes makes it an attractive candidate for bioremediation of petroleum sludge as n-alkanes are the principal components of petroleum sludge.

When studying the growth kinetics of microbes on toxic compounds, the classical Monod kinetics is inadequate to estimate the kinetic parameters. Instead, Haldane’s model integrates substrate inhibition to the standard model and has been found to be generally accurate in modeling the kinetics of growth inhibiting substrate (Arif *et al*. 2013). A simple hydrocarbon such as phenol poses a lesser cellular machinery degrading activity than the complex concoction of toxic compounds in petroleum sludge. Growth on phenol has been shown to produce a maximum growth rate as high as 0.542 hr^−1^ for an efficient strain (Arif *et al*. 2013). In petroleum sludge, a lower growth rate is caused by simultaneous activities that covers assimilatory and detoxification that include enzymatic degradation, membrane permeability modifications, and pumping machineries which consumes energy and reduces growth yield. A literature search showed that there are limited data on the biodegradation kinetics of petroleum sludge. The maximum specific growth rate is considered high if compared to a μmax of 0.012 hr^−1^ for *Pseudomonas* sp. strain LP1 (Obayori *et al*. 2009) and a μmax of 0.021 hr^−1^ for a petrophilic consortia (Helmy *et al*. 2009). The *K_i_* value is higher than *Neosartorya* sp. BL4 with a *K_i_* value of 1,860 mg/L (Yi *et al*. 2011) and petrophilic consortia with a value of 2,738 mg/L (Helmy *et al*. 2009). The high inhibition constant indicates a better adaptation and higher tolerance towards TPH by strain ZASH. Under an extreme toxic environment, growth is secondary to elimination of toxic compounds. The absence of growth and the presence of degradation activity in strain ZASH at 4% (w/v) TPH reflect this phenomenon. The strain is directing its cellular machinery for petroleum sludge detoxification instead of growth. The maximum degradation rate for a complex hydrocarbon composition such as petroleum sludge is low when compared to the biodegradation rates of diesel, a simpler hydrocarbon mixture that could be as high as 71.95 mg/L hr^−1^ (Hong *et al*. 2010). In addition, petroleum sludge contains microbial-inhibiting agents, such as heavy metals, and higher percentages of toxic aromatic compounds than diesel (Y. Wang *et al*. 2017).

**Figure 14:**
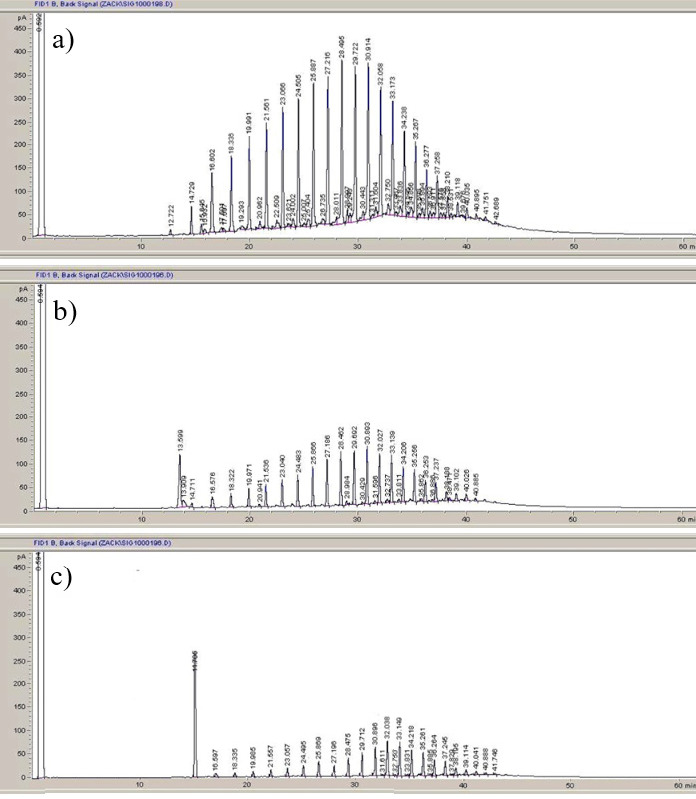
Gas chromatogram of biodegradation hydrocarbons in petroleum sludge at the initial concentration of 1% (w/v) with *Methylobacterium* sp. strain ZASH. (a) Control sample after 15 days of incubation; (b) Sample after 15 days of incubation with *Methylobacterium* sp. strain ZASH; and (c) Sample after 15 days of incubation with *Methylobacterium* sp. strain ZASH and 5% (w/v) of sawdust.

The addition of sawdust did not improve the bacterial degradation of hydrocarbons, but it helped to remove the undegraded hydrocarbons. This evidence can be seen in the GC chromatogram shown in Fig. 14 where removal of hydrocarbon components can be seen after 15 days of incubation with or without sawdust addition. The ability of sawdust to enhance the removal of hydrocarbons has long been studied by several authors (Ismail *et al*. 2019; Tanee & Jude 2017). The result of this study shows that the addition of sawdust improves hydrocarbon degradation dramatically with almost 100% removal. Upon further examination, we discovered that there is no additional benefit in sawdust addition especially in liquid cultures as the improved degradation observed upon the addition of sawdust was due to hydrocarbon absorption by the sawdust. Previous works that reported enhancement of degradation activity of sawdust addition did not reveal the extent of hydrocarbon absorption by the sawdust (Ismail *et al*. 2019; Tanee & Jude 2017). The bioavailability of the hydrocarbon after absorption is reduced. However, sawdust addition to oil-contaminated sludge is likely an important strategy as it is reported to increase in remediation efficiency (Huang *et al*. 2019; Ismail *et al*. 2019; Tanee & Jude 2017) for reasons previously discussed.

## CONCLUSION

In summary, we have discovered a *Methylobacterium* sp. strain that degrades various ranges of hydrocarbons in petroleum sludge with carbon numbers from C-12 to C-36. This strain was able to degrade up to 70% of 1% (w/v) petroleum sludge in 15 days. The ability of the strain to degrade petroleum sludge under a broad optimal temperature range that covers subtropical and tropical areas is an advantage for bioremediation programmes using this strain. Although sawdust did not enhance biodegradation in the liquid culture, we are currently exploring oil biodegradation studies using sawdust as a bulking agent.

## Figures and Tables

**Figure 11 f11-tlsr-34-2-197:**
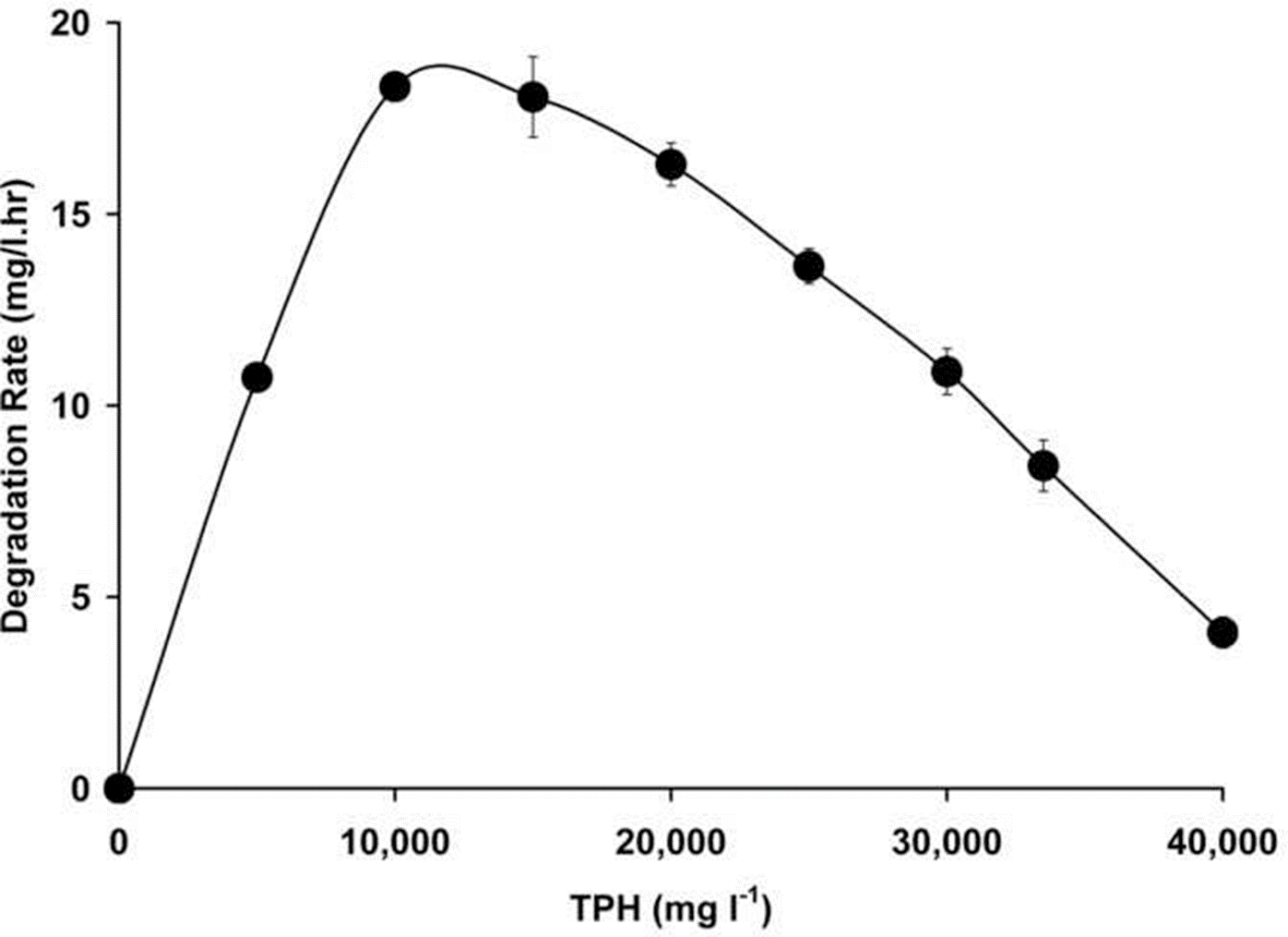
Biodegradation rates at different TPH concentrations. Each data point represents mean ± SD (*n* = 3).

## References

[b1-tlsr-34-2-197] Abarian M, Hassanshahian M, Esbah A (2019). Degradation of phenol at high concentrations using immobilization of Pseudomonas putida P53 into sawdust entrapped in sodium-alginate beads. Water Science and Technology.

[b2-tlsr-34-2-197] AL-Doury MMI (2019). Treatment of oily sludge produced from Baiji oil refineries using surfactants. Petroleum Science and Technology.

[b3-tlsr-34-2-197] Alhefeiti MA, Athamneh K, Vijayan R, Ashraf SS (2021). Bioremediation of various aromatic and emerging pollutants by Bacillus cereus sp. isolated from petroleum sludge. Water Science and Technology.

[b4-tlsr-34-2-197] Ali N, Eliyas M, Al-Sarawi H, Radwan SS (2011). Hydrocarbon-utilizing microorganisms naturally associated with sawdust. Chemosphere.

[b5-tlsr-34-2-197] Arif NM, Ahmad SA, Syed MA, Shukor MY (2013). Isolation and characterization of a phenol-degrading *Rhodococcus* sp. strain AQ5NOL 2 KCTC 11961BP. Journal of Basic Microbiology.

[b6-tlsr-34-2-197] Behera ID, Basak G, Kumar RR, Sen R, Meikap BC (2020). Treatment of petroleum refinery sludge by petroleum degrading bacterium *Stenotrophomonas pavanii* IRB19 as an efficient novel technology. Journal of Environmental Science and Health, Part A.

[b7-tlsr-34-2-197] Cecotti M, Coppotelli BM, Mora VC, Viera M, Morelli IS (2018). Efficiency of surfactant-enhanced bioremediation of aged polycyclic aromatic hydrocarbon-contaminated soil: Link with bioavailability and the dynamics of the bacterial community. Science of the Total Environment.

[b8-tlsr-34-2-197] Chen L, Lei Z, Luo X, Wang D, Li L, Li A (2019). Biological degradation and transformation characteristics of total petroleum hydrocarbons by oil degradation bacteria adsorbed on modified straw. ACS Omega.

[b9-tlsr-34-2-197] Cheng T, Liang J, He J, Hu X, Ge Z, Liu J (2017). A novel rhamnolipid-producing Pseudomonas aeruginosa ZS1 isolate derived from petroleum sludge suitable for bioremediation. AMB Express.

[b10-tlsr-34-2-197] Devi MP, Reddy MV, Juwarkar A, Sarma PN, Mohan SRV (2011). Effect of co-culture and nutrients supplementation on bioremediation of crude petroleum sludge. Clean–Soil, Air, Water.

[b11-tlsr-34-2-197] Effendi AJ, Kamath R, McMillen S, Sihota N, Zuo E, Sra K, Kong D, Wisono T, Syakir J (2017). Strategies for enhancing bioremediation for hydrocarbon-impacted soils.

[b12-tlsr-34-2-197] Felsenstein J (1985). Confidence limits on phylogenies: An approach using the bootstrap. Evolution.

[b13-tlsr-34-2-197] Habib S, Ahmad SA, Johari WLW, Abd Shukor MY, Alias SA, Khalil KA, Yasid NA (2018). Evaluation of conventional and response surface level optimisation of n-dodecane (n-C12) mineralisation by psychrotolerant strains isolated from pristine soil at Southern Victoria Island, Antarctica. Microbial Cell Factories.

[b14-tlsr-34-2-197] Hamidi Y, Ataei SA, Sarrafi A (2021). A highly efficient method with low energy and water consumption in biodegradation of total petroleum hydrocarbons of oily sludge. Journal of Environmental Management.

[b15-tlsr-34-2-197] Hamzah A, Phan C-W, Abu Bakar NF, Wong K-K (2013). Biodegradation of crude oil by constructed bacterial consortia and the constituent single bacteria isolated from Malaysia. Bioremediation Journal.

[b16-tlsr-34-2-197] Hamzah A, Zarin MA, Hamid AA, Omar O, Senafi S (2012). Optimal physical and nutrient parameters for growth of Trichoderma virens UKMP-1M for heavy crude oil degradation. Sains Malaysiana.

[b17-tlsr-34-2-197] Helmy Q, Kardena E, Wisjnuprapto (2009). Performance of petrofilic consortia and effect of surfactant tween 80 addition in the oil sludge removal process. Journal of Applied Sciences in Environmental Sanitation.

[b18-tlsr-34-2-197] Hong S-H, Kim J-Y, Cho K-S (2010). Isolation and characterization of a diesel-degrading bacterium, Gordonia sp. SD8. Microbiology and Biotechnology Letters.

[b19-tlsr-34-2-197] Huang Y, Pan H, Wang Q, Ge Y, Liu W, Christie P (2019). Enrichment of the soil microbial community in the bioremediation of a petroleum-contaminated soil amended with rice straw or sawdust. Chemosphere.

[b20-tlsr-34-2-197] Islam B (2015). Petroleum sludge, its treatment and disposal: A review. International Journal of Chemical Sciences.

[b21-tlsr-34-2-197] Ismail AS, El-Sheshtawy HS, Khalil NM (2019). Bioremediation process of oil spill using fatty-lignocellulose sawdust and its enhancement effect. Egyptian Journal of Petroleum.

[b22-tlsr-34-2-197] Ji L, Fu X, Wang M, Xu C, Chen G, Song F, Guo S, Zhang Q (2019). Enzyme cocktail containing NADH regeneration system for efficient bioremediation of oil sludge contamination. Chemosphere.

[b23-tlsr-34-2-197] Johnson OA, Affam AC (2019). Petroleum sludge treatment and disposal: A review. Environmental Engineering Research.

[b24-tlsr-34-2-197] Jukes TH, Cantor CR (1969). Evolution of protein molecules. Mammalian Protein Metabolism.

[b25-tlsr-34-2-197] Kaczorek E, Pacholak A, Zdarta A, Smułek W (2018). The impact of biosurfactants on microbial cell properties leading to hydrocarbon bioavailability increase. Colloids and Interfaces.

[b26-tlsr-34-2-197] Lee GLY, Ahmad SA, Yasid NA, Zulkharnain A, Convey P, Johari WLW, Alias SA, Gonzalez-Rocha G, Shukor MY (2018). Biodegradation of phenol by cold-adapted bacteria from Antarctic soils. Polar Biology.

[b27-tlsr-34-2-197] Lin C, Cheruiyot NK, Hoang H-G, Le T-H, Tran H-T, Bui X-T (2021). Benzophenone biodegradation and characterization of malodorous gas emissions during co-composting of food waste with sawdust and mature compost. Environmental Technology & Innovation.

[b28-tlsr-34-2-197] Liu Y, Wan YY, Wang C, Ma Z, Liu X, Li S (2020). Biodegradation of n-alkanes in crude oil by three identified bacterial strains. Fuel.

[b29-tlsr-34-2-197] Margush T, McMorris FR (1981). Consensus n-trees. Bulletin of Mathematical Biology.

[b30-tlsr-34-2-197] Mishra S, Jyot J, Kuhad RC, Lal B (2001). In situ bioremediation potential of an oily sludge-degrading bacterial consortium. Current Microbiology.

[b31-tlsr-34-2-197] Muliadi FNA, Halmi MIE, Wahid SBA, Gani SSA, Zaidan UH, Mahmud K, Abd Shukor MY (2021). Biostimulation of microbial communities from Malaysian agricultural soil for detoxification of metanil yellow dye: A response surface methodological approach. Sustainability.

[b32-tlsr-34-2-197] Nikitina EV, Yakusheva OI, Zaripov SA, Galiev RA, Garusov AV, Naumova RP (2003). Distribution and physiological state of microorganisms in petrochemical oily sludge. Microbiology.

[b33-tlsr-34-2-197] Obayori OS, Ilori MO, Adebusoye SA, Oyetibo GO, Omotayo AE, Amund OO (2009). Degradation of hydrocarbons and biosurfactant production by Pseudomonas sp. strain LP1. World Journal of Microbiology and Biotechnology.

[b34-tlsr-34-2-197] Ossai IC, Ahmed A, Hassan A, Hamid FS (2020). Remediation of soil and water contaminated with petroleum hydrocarbon: A review. Environmental Technology & Innovation.

[b35-tlsr-34-2-197] Page RDM (1996). Tree View: An application to display phylogenetic trees on personal computers. Bioinformatics.

[b36-tlsr-34-2-197] Pugazhendi A, Abbad Wazin H, Qari H, Basahi JMA-B, Godon JJ, Dhavamani J (2017). Biodegradation of low and high molecular weight hydrocarbons in petroleum refinery wastewater by a thermophilic bacterial consortium. Environmental Technology.

[b37-tlsr-34-2-197] Rahman KSM, Rahman TJ, Banat IM, Lord R, Street G, Singh SN, Tripathi RD (2007). Bioremediation of petroleum sludge using bacterial consortium with biosurfactant. Environmental bioremediation technologies.

[b38-tlsr-34-2-197] Roleda MY, Hurd CL (2019). Seaweed nutrient physiology: Application of concepts to aquaculture and bioremediation. Phycologia.

[b39-tlsr-34-2-197] Sabullah MK, Rahman MF, Ahmad SA, Sulaiman MR, Shukor MS, Shamaan NA, Shukor MY (2017). Assessing resistance and bioremediation ability of Enterobacter sp. strain saw-1 on molybdenum in various heavy metals and pesticides. Journal of Mathematical and Fundamental Sciences.

[b40-tlsr-34-2-197] Saeedi M, Li LY, Grace JR (2019). Simultaneous removal of polycyclic aromatic hydrocarbons and heavy metals from natural soil by combined non-ionic surfactants and EDTA as extracting reagents: Laboratory column tests. Journal of Environmental Management.

[b41-tlsr-34-2-197] Saitou N, Nei M (1987). The neighbor-joining method: A new method for reconstructing phylogenetic trees. Molecular Biology and Evolution.

[b42-tlsr-34-2-197] Sarkar J, Kazy SK, Gupta A, Dutta A, Mohapatra B, Roy A, Bera P, Mitra A, Sar P (2016). Biostimulation of indigenous microbial community for bioremediation of petroleum refinery sludge. Frontiers in Microbiology.

[b43-tlsr-34-2-197] Shaban SM, Kim D-H (2020). The influence of the Gemini surfactants hydrocarbon tail on *in-situ* synthesis of silver nanoparticles: Characterization, surface studies and biological performance. Korean Journal of Chemical Engineering.

[b44-tlsr-34-2-197] Shivanand P, Matussi NBAB, Lim LH, Kumar V (2021). Microbial surfactants: Current perspectives and role in bioremediation. Rhizomicrobiome Dynamics in Bioremediation.

[b45-tlsr-34-2-197] Shukor MY, Rahman MF, Shamaan NA, Syed MA (2009). Reduction of molybdate to molybdenum blue by *Enterobacter* sp. strain Dr. Y13. Journal of Basic Microbiology.

[b46-tlsr-34-2-197] Shuler ML, Kargi F (1992). Bioprocess engineering.

[b47-tlsr-34-2-197] Somtrakoon K, Chouychai W (2019). Effect of Triton X-100 and Tween 80 on removal of polycyclic aromatic hydrocarbons and possibility of cadmium accumulation by Siam weed (*Chromolaena odorata*). Songklanakarin Journal of Science & Technology.

[b48-tlsr-34-2-197] Srivastva N, Vishwakarma P, Bhardwaj Y, Singh A, Manjunath K, Dubey SK (2017). Kinetic and molecular analyses reveal isoprene degradation potential of *Methylobacterium* sp. Bioresource Technology.

[b49-tlsr-34-2-197] Suganthi SH, Murshid S, Sriram S, Ramani K (2018). Enhanced biodegradation of hydrocarbons in petroleum tank bottom oil sludge and characterization of biocatalysts and biosurfactants. Journal of Environmental Management.

[b50-tlsr-34-2-197] Tanee FBG, Jude K (2017). Effect of detergent and sawdust addition on hydrocarbon reduction and growth of *Abelmoschus esculentus* L. (Okra) in a petroleum-contaminated soil. Nigerian Journal of Biotechnology.

[b51-tlsr-34-2-197] Thompson JD, Higgins DG, Gibson TJ (1994). CLUSTAL W: Improving the sensitivity of progressive multiple sequence alignment through sequence weighting, position-specific gap penalties and weight matrix choice. Nucleic Acids Research.

[b52-tlsr-34-2-197] Wang D, Lin J, Lin J, Wang W, Li S (2019). Biodegradation of petroleum hydrocarbons by Bacillus subtilis BL-27, a strain with weak hydrophobicity. Molecules.

[b53-tlsr-34-2-197] Wang G, Yin Q, Shen J, Bai Y, Ma X, Du Z, Wang W (2017). Surface activities and aggregation behaviors of cationic-anionic fluorocarbon-hydrocarbon surfactants in dilute solutions. Journal of Molecular Liquids.

[b54-tlsr-34-2-197] Wang Y, Zhang X, Pan Y, Chen Y (2017). Analysis of oil content in drying petroleum sludge of tank bottom. International Journal of Hydrogen Energy.

[b55-tlsr-34-2-197] Wongsa P, Tanaka M, Ueno A, Hasanuzzaman M, Yumoto I, Okuyama H (2004). Isolation and characterization of novel strains of *Pseudomonas aeruginosa* and *Serratia marcescens* possessing high efficiency to degrade gasoline, kerosene, diesel oil, and lubricating oil. Current Microbiology.

[b56-tlsr-34-2-197] Xia M, Fu D, Chakraborty R, Singh RP, Terry N (2019). Enhanced crude oil depletion by constructed bacterial consortium comprising bioemulsifier producer and petroleum hydrocarbon degraders. Bioresource Technology.

[b57-tlsr-34-2-197] Xu X, Liu W, Wang W, Tian S, Jiang P, Qi Q, Li F, Li H, Wang Q, Li H (2019). Potential biodegradation of phenanthrene by isolated halotolerant bacterial strains from petroleum oil polluted soil in Yellow River Delta. Science of The Total Environment.

[b58-tlsr-34-2-197] Yan Z, Song N, Cai H, Tay J-H, Jiang H (2012). Enhanced degradation of phenanthrene and pyrene in freshwater sediments by combined employment of sediment microbial fuel cell and amorphous ferric hydroxide. Journal of Hazardous Materials.

[b59-tlsr-34-2-197] Yang J, Zhang C-T, Yuan X-J, Zhang M, Mo X-H, Tan L-L, Zhu L-P, Chen W-J, Yao M-D, Hu B (2018). Metabolic engineering of *Methylobacterium* extorquens AM1 for the production of butadiene precursor. Microbial Cell Factories.

[b60-tlsr-34-2-197] Yi T, Lee E-H, Park H, Cho K-S (2011). Biodegradation of petroleum hydrocarbons by Neosartorya sp. BL4. Journal of Environmental Science and Health, Part A.

[b61-tlsr-34-2-197] Zhao G, Sheng Y, Wang C, Yang J, Wang Q, Chen L (2018). *In situ* microbial remediation of crude oil-soaked marine sediments using zeolite carrier with a polymer coating. Marine Pollution Bulletin.

